# Phosphatase Inhibitors Function as Novel, Broad Spectrum Botulinum Neurotoxin Antagonists in Mouse and Human Embryonic Stem Cell-Derived Motor Neuron-Based Assays

**DOI:** 10.1371/journal.pone.0129264

**Published:** 2015-06-10

**Authors:** Erkan Kiris, Jonathan E. Nuss, Stephanie M. Stanford, Laura M. Wanner, Lisa Cazares, Michael F. Maestre, Hao T. Du, Glenn Y. Gomba, James C. Burnett, Rick Gussio, Nunzio Bottini, Rekha G. Panchal, Christopher D. Kane, Lino Tessarollo, Sina Bavari

**Affiliations:** 1 Geneva Foundation, Tacoma, WA, United States of America; 2 Department of Molecular and Translational Sciences, US Army Medical Research Institute of Infectious Diseases, Frederick, MD, United States of America; 3 Mouse Cancer Genetics Program, Center for Cancer Research, National Cancer Institute (NCI), Frederick, MD, United States of America; 4 Division of Cellular Biology, La Jolla Institute for Allergy and Immunology, La Jolla, CA, United States of America; 5 Leidos Biomedical Research, Inc., Computational Drug Development Group (CDDG), NCI, Frederick, MD, United States of America; 6 CDDG, Developmental Therapeutics Program, NCI, Frederick, MD, United States of America; 7 Henry M. Jackson Foundation, Bethesda, MD, United States of America; 8 DoD Biotechnology High Performance Computing Software Applications Institute (BHSAI), Telemedicine and Advanced Technology Research Center (TATRC), US Army Medical Research and Materiel Command (USAMRMC), Frederick, MD, United States of America; University of Wisconsin, Food Research Institute, UNITED STATES

## Abstract

There is an urgent need to develop novel treatments to counter Botulinum neurotoxin (BoNT) poisoning. Currently, the majority of BoNT drug development efforts focus on directly inhibiting the proteolytic components of BoNT, i.e. light chains (LC). Although this is a rational approach, previous research has shown that LCs are extremely difficult drug targets and that inhibiting multi-serotype BoNTs with a single LC inhibitor may not be feasible. An alternative approach would target neuronal pathways involved in intoxication/recovery, rather than the LC itself. Phosphorylation-related mechanisms have been implicated in the intoxication pathway(s) of BoNTs. However, the effects of phosphatase inhibitors upon BoNT activity in the physiological target of BoNTs, i.e. motor neurons, have not been investigated. In this study, a small library of phosphatase inhibitors was screened for BoNT antagonism in the context of mouse embryonic stem cell-derived motor neurons (ES-MNs). Four inhibitors were found to function as BoNT/A antagonists. Subsequently, we confirmed that these inhibitors protect against BoNT/A in a dose-dependent manner in human ES-MNs. Additionally, these compounds provide protection when administered in post-intoxication scenario. Importantly, the inhibitors were also effective against BoNT serotypes B and E. To the best of our knowledge, this is the first study showing phosphatase inhibitors as broad-spectrum BoNT antagonists.

## Introduction

Botulinum neurotoxins (BoNTs), the causative agents of the life-threatening disease botulism, are among the most toxic biological substances known to man [1]. Despite their remarkably high toxicity, BoNTs are used as therapeutics to treat a range of medical conditions characterized by excessive muscle tone including ophthalmologic, urogenital, and dermatologic disorders. Four commercial products containing either BoNT/A or BoNT/B have been approved by the FDA (Botox, Myobloc, Dysport, and Xeomin) and are most commonly used for the cosmetic treatment of facial wrinkles [2]. According to the 2013 statistics from American Society of Plastic Surgeons, BoNT treatment is the top non-surgical cosmetic procedure (about 6.3 million procedures in 2013) and its usage has increased 703% in the past 13 years [3]. Consequently, there are concerns about accidental overdosing in clinics in addition to unintentional poisoning through contaminated food or liquids [4]. Furthermore, these toxins are among the CDC’s highest priority biothreat agents because of their relative ease of production and high toxicity. In fact, BoNTs have been weaponized [5], consequently there are heightened concerns over potential malicious uses of these toxins. Currently, mechanical ventilation is the only life-saving option once the BoNTs are internalized into motor neurons and paralysis is manifested. FDA approved antitoxins are available for the treatment of botulism; however antibody therapies can only neutralize the fraction of toxin within the vasculature and therefore must be administered prior to neuronal uptake and intoxication in order to be effective [6, 7]. However, even with the antibody therapy, prolonged mechanical respiration may be necessary as BoNTs can persist in neurons for up to several months [8]. Such long-term care would be unfeasible for treating a modest outbreak or bioterror event given the limited infrastructure and the associated cost, which can be as high as $350,000 for two weeks of treatment for each patient [4].

Currently, there are no small molecule therapeutics to treat BoNT poisoning after neuronal intoxication. Most of BoNT drug development efforts have focused on inhibiting the proteolytic activity of the light chain (LC) [9, 10]. Despite extensive research on LC inhibitors [11–14], there is no compelling evidence that these compounds will provide meaningful *in vivo* protection in a post-exposure scenario. In addition, these approaches mostly target a single serotype (BoNT/A). However, BoNT/A is responsible for only half of the human botulism cases and BoNT serotypes B and E also pose significant threats [15]. Therefore, novel approaches are needed to develop therapeutically viable countermeasures that are effective against multiple BoNT serotypes.

An alternative strategy would focus on the modulation of cellular pathways that are involved in intoxication and/or recovery. Such neuronal pathways can potentially provide novel drug targets with potential for treating botulism. Generally speaking, the sequence of events during BoNT intoxication are well understood [16]. However, the understanding of host factor response to BoNT intoxication and the neuronal signaling nodes that are impacted by BoNT-mediated inhibition of neuroexocytosis are poorly comprehended. Importantly, previous research implicated certain neuronal pathways that may be modulated by BoNT exposure. For example, it has been shown that BoNT intoxication induces axonal sprouting [17–19]. Axonal sprouting is a complex event resulting in extensive morphological changes and requires activation of certain neuronal pathways to modulate cytoskeletal re-modeling. Molecular mechanisms underlying the neuronal events mediating this process in response to BoNT exposure remain largely unknown. Given that the persistence of botulism symptoms and BoNT clearance from neurons is generally a slow process and can vary from days to months depending upon the BoNT serotype, modulation of host pathways that are involved or responsive to BoNT intoxication may support the development of therapeutics that counter BoNTs and restore the connectivity between the motor neurons and myocytes.

Previous studies suggest that phosphorylation-related cellular processes may play critical role(s) in regulating BoNT activity in cells. For example, it has been suggested that LCs are substrates for Src kinases and that toxin phosphorylation may influence its activity and protein half-life [20–24]. Additionally, small molecule Src Family Kinase (SFK) inhibitors exhibit efficacy against BoNT serotypes A, B and E in a dose-dependent manner in human ES-cell derived motor neurons (ES-MNs) [25]. However, the effect(s) of phosphatase inhibitors upon BoNT mediated soluble N-ethylmaleimide-sensitive factor attachment protein receptor (SNARE) cleavage following intoxication has not been explored. It is established that proper activity of both kinases and phosphatases are required for normal cellular homeostasis. Indeed, aberrant phosphorylation is associated with many neurodegenerative, inflammatory, and oncologic diseases. Therefore protein kinases and more recently phosphatases have been the focus of significant research as potential targets for drug discovery [26].

In this study, we sought to determine whether well-characterized phosphatase inhibitors would impact BoNT intoxication in motor neurons. Both mouse and human ES cell-derived motor neuron systems were used for these studies as both models are exquisitely sensitive to intoxication by multiple BoNTs and faithfully recapitulate primary motor neurons [27–29]. Here we have screened a small library of known phosphatase inhibitors using motor neurons, cells normally targeted by BoNTs. Using mouse embryonic stem cell-derived motor neurons (mES-MNs) as a screening platform [30], we identified 4 phosphatase inhibitors as novel BoNT/A antagonists. Subsequently, we confirmed that these inhibitors protected SNARE proteins against BoNT/A, /B and /E in human embryonic stem cell-derived motor neurons (hES-MNs) in a dose-dependent manner. Importantly, these compounds provided protection against these three serotypes when administrated in a post intoxication model. To the best of our knowledge, this is the first study demonstrating the ability of phosphatase inhibitors to antagonize multiple BoNT serotypes in both pre- and post-intoxication scenarios using cellular models of both murine and human motor neurons.

## Materials and Methods

### Directed differentiation of mouse and human ES cells into motor neurons

Mouse ES cells (HBG3 cell line) in which eGFP expression is driven by mouse motor neuron specific transcription factor Hb9 promoter, were cultured and differentiated to spinal motor neurons as previously described [28, 30]. H9 human ES cells (WA09) were purchased from WiCell Research Institute, and were cultured and differentiated as described previously [31–33]. Both systems are highly sensitive to BoNT intoxication and can be utilized to examine and characterize potential multi-serotype BoNT inhibitors [25, 30].

### Determination of BoNT/A activity in motor neuron-based assays using BoNT/A cleavage sensitive antibodies

Li-Cor assay and analysis were performed as described previously [34]. Briefly, mouse ES-MNs were cultured on 96-well plates (BD Falcon Imaging Microplates) and intoxicated with 250 pM BoNT/A for 3 hr. Compounds (20μM) from Enzo Life Sciences phosphatase inhibitor library were added to the cultures 30 min prior to BoNT intoxication. SNAP-25 protein cleavage was detected using standard immunofluorescence procedures employing total SNAP-25 antibody (N-terminal specific antibody, SMI-81, Covance) and full length SNAP-25 antibodies (BoNT/A cleavage sensitive [BACS] F2070 antibodies) [34, 35]. Generation and characterization of BACS antibodies have been published [27, 34]. The Li-Cor imaging assay was performed using a Li-Cor odyssey infrared imaging system (Li-Cor, Lincoln, NE), as described previously [34].

### BoNT intoxication and western blot analyses to measure SNARE protein cleavage

**Fig 1:**
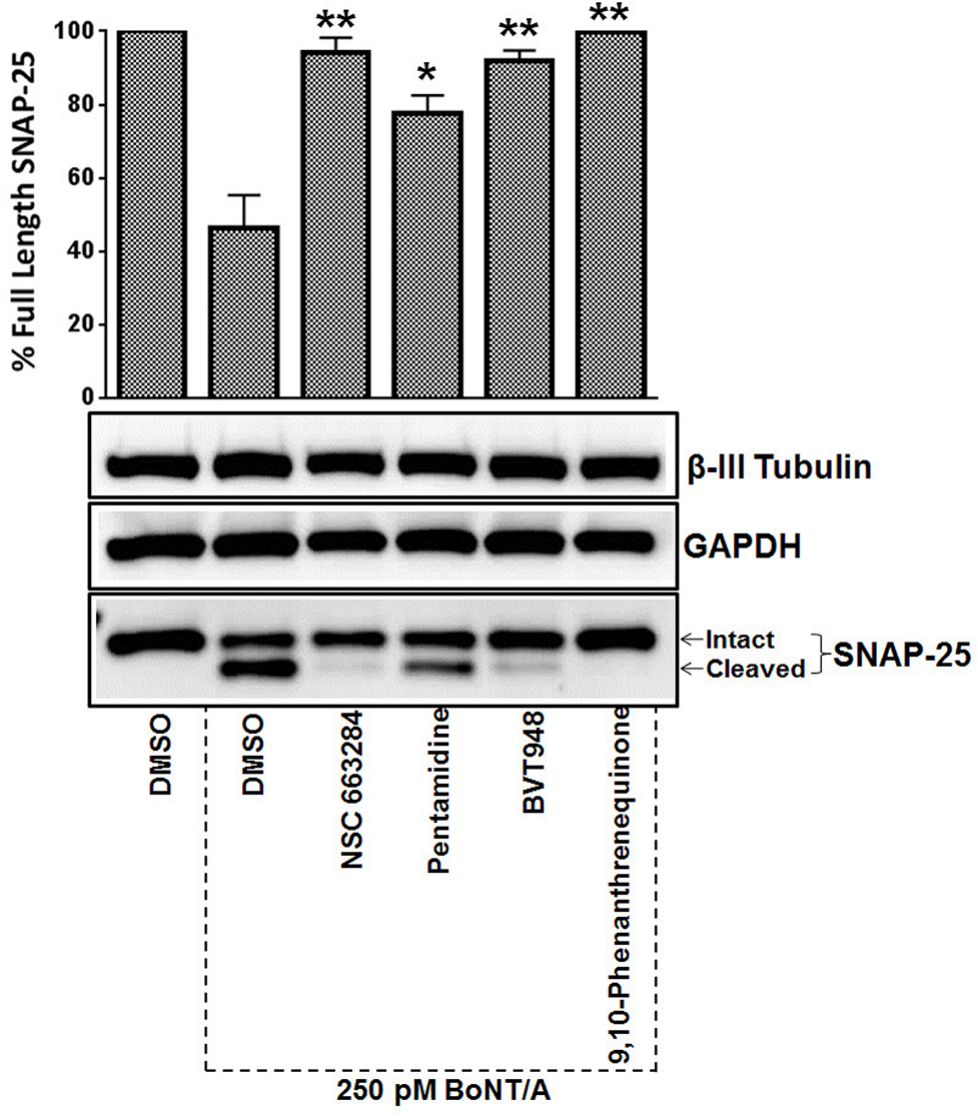
Mouse ES-derived motor neuron based assays identify phosphatase inhibitors as BoNT/A antagonists. Four phosphatase inhibitors identified as potential BoNT inhibitors in imaging based assays were analyzed in secondary screens using mES-MNs to confirm their protective activities against BoNT/A. mES-MNs were incubated with 20 μM compound for 30 minutes and then treated with 250 pM BoNT/A for 4 hrs. BoNT/A activity was measured by Western blot analysis and band densitometry. GAPDH and neuron specific β-III tubulin (Tuj1) served as loading controls. Error bars represent standard error of means (n = 3). ★★, ★ Value significant at 99% and 95% confidence level, respectively, compared to DMSO+Toxin control conditions (Student’s *t-*test).

**Fig 2:**
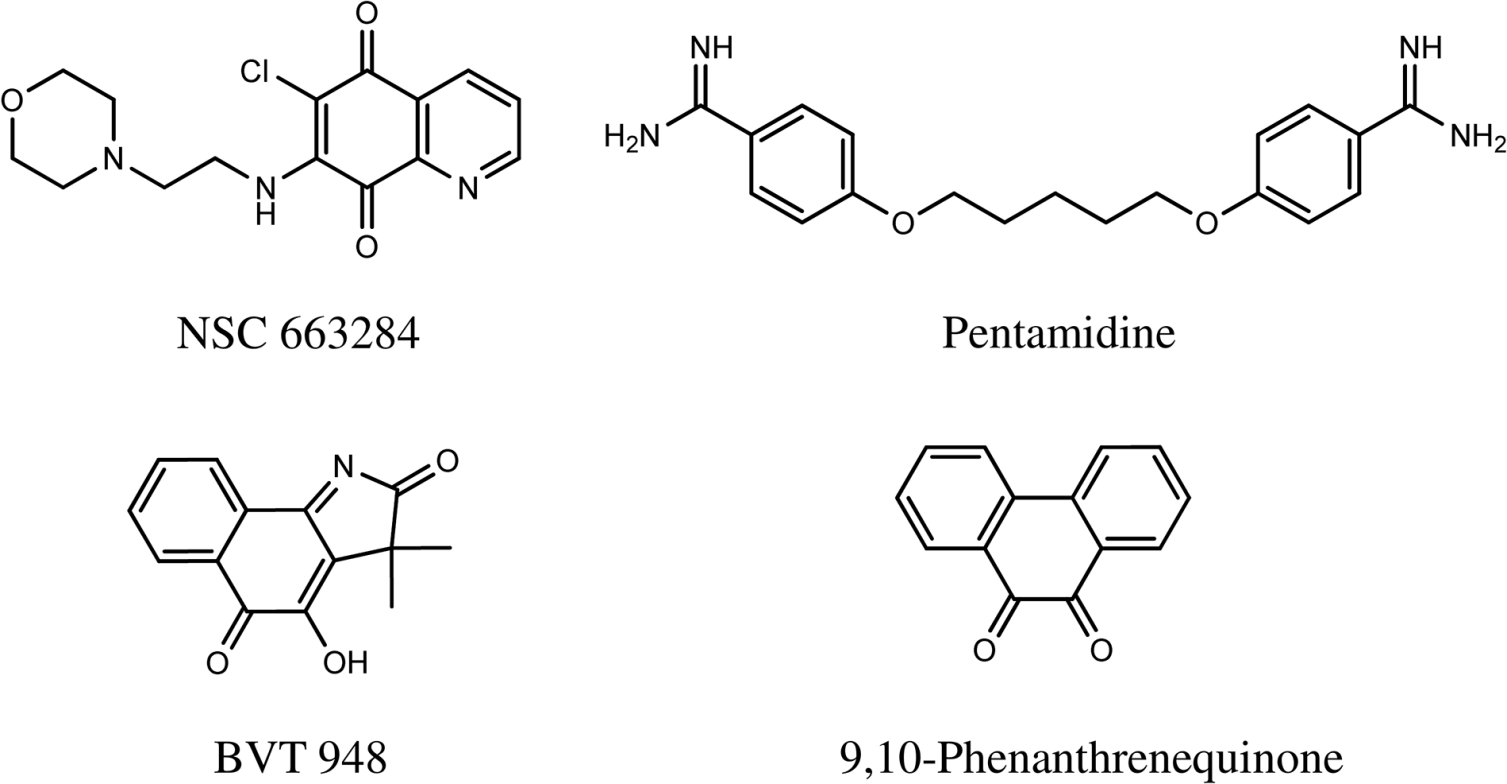
The chemical names and structures of the identified compounds.

**Fig 3:**
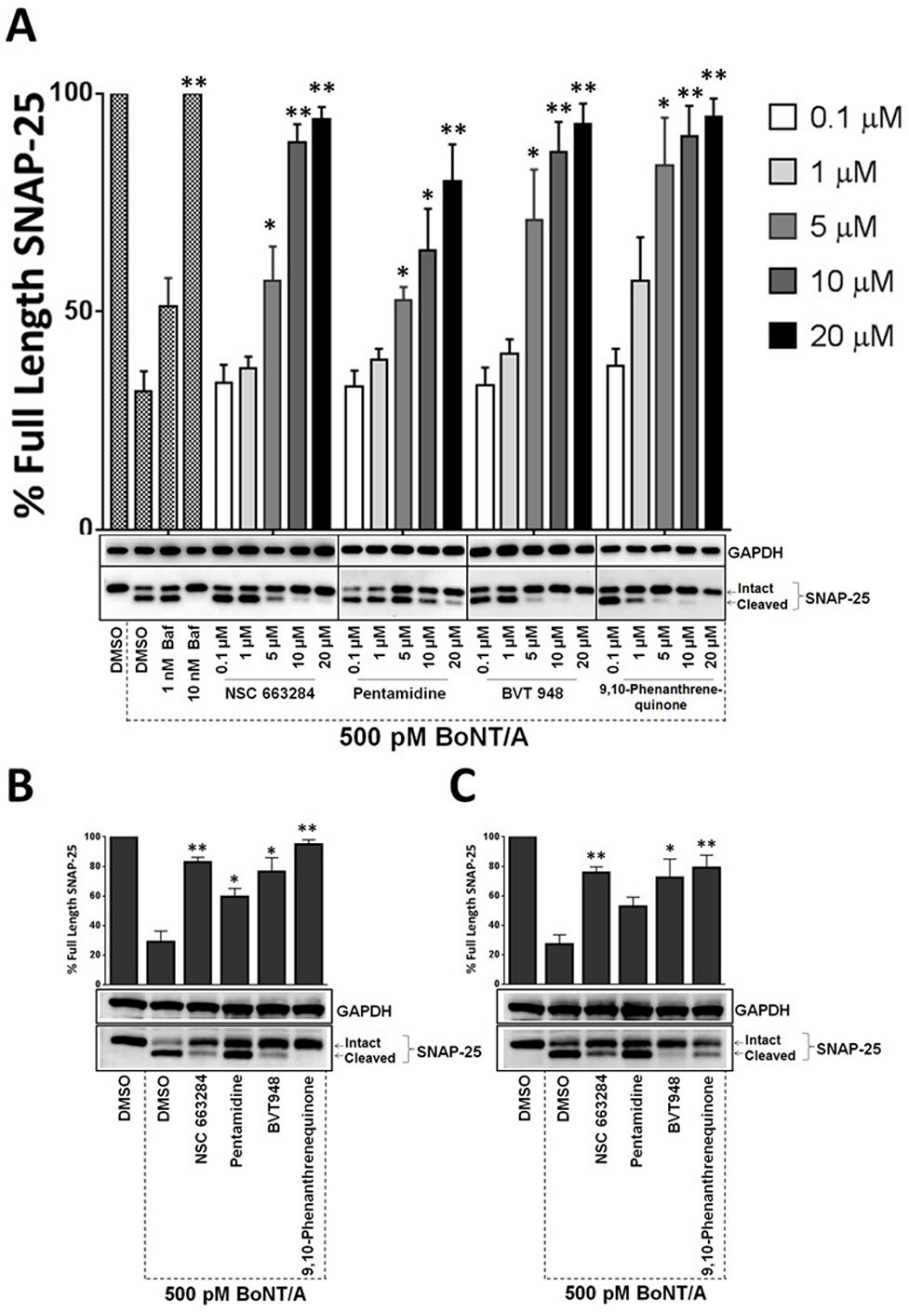
Efficacies of the phosphatase inhibitors against BoNT/A were validated using human ES-MNs. Four phosphatase inhibitors were evaluated for their ability to protect SNAP-25 in hES-MNs. Cells were treated with increasing concentrations (0.1–20 μM) of inhibitors for 30 minutes and then incubated with 500 pM BoNT/A for 4 hrs (A). Cell lysates were then prepared and analyzed by Western blotting using SNAP-25 antibodies. Bafilomycin was used as a control compound and GAPDH served as a loading control. Efficacies of the inhibitors (20 μM) against 500 pM BoNT/A 30 min (B) and 60 min (C) post-intoxication. The values are given as mean±SEM from at least three independent experiments. ★★, ★ Value significant at 99% and 95% confidence level, respectively, compared to DMSO+Toxin control conditions (Student’s *t-*test).

**Fig 4:**
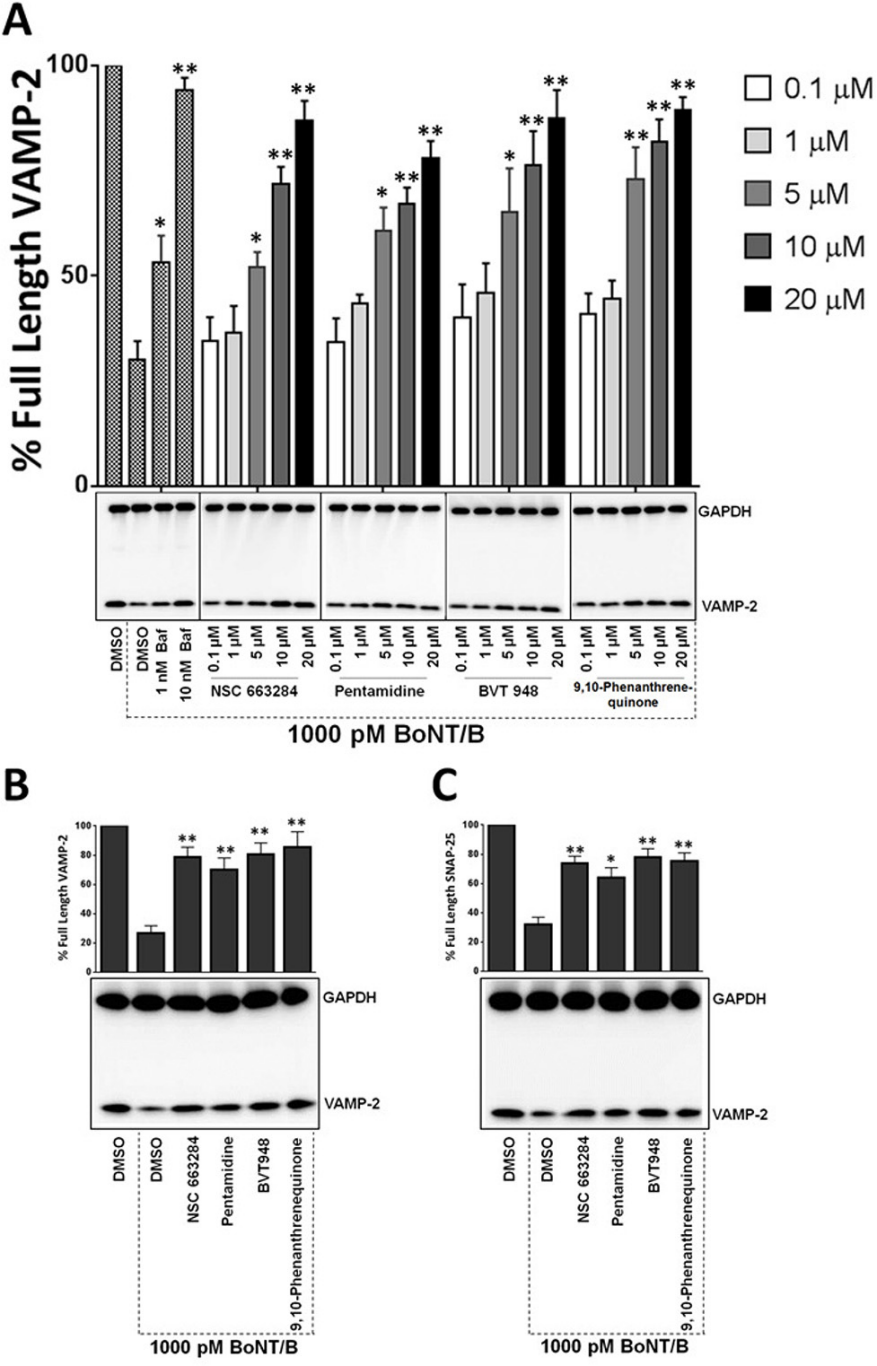
Phosphatase inhibitors protect against BoNT/B in a dose-dependent manner. (A) hES-MNs were treated with increasing concentrations of compounds ranging from 0.1 μM to 20 μM for 30 min and then intoxicated with BoNT/B. Bafilomycin served as a control compound. The inhibitors antagonize BoNT/B in 30 (B) and 60 min (C) post-exposure human neuron models. GAPDH and VAMP-2 antibodies were applied to the blots simultaneously. The values are mean ± SEM, calculated from at least three independent experiments. ★★, ★ Value significant at 99% and 95% confidence levels, respectively, compared to control conditions (Student’s *t-*test).

**Fig 5:**
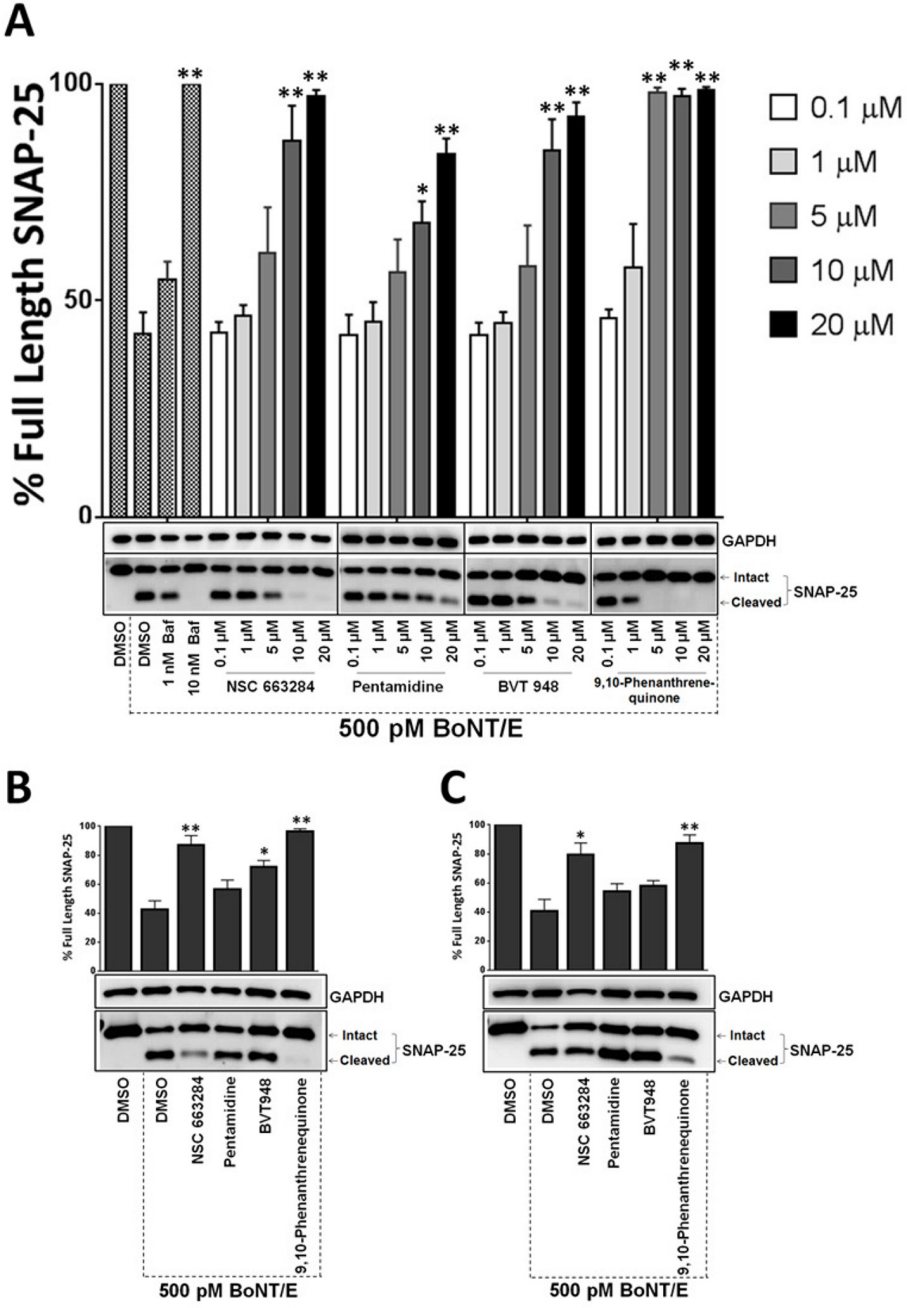
Phosphatase inhibitors antagonize BoNT/E in hES-MNs in both pre and post-intoxication models. (A) Compounds (0.1–20 μM) were added to hES-MNs, intoxicated with activated BoNT/E (500 pM) and analyzed by Western blotting. The compounds inhibit BoNT/E in a post-exposure human neuron model (B and C, similar to Figs 3 and 4). Compounds (20μM) were added either 30 min (B) or 60 min (C) after BoNT/E (500 pM) intoxication. The values are presented as mean±SEM from at least three independent experiments. ★★, ★ Value significant at 99% and 95% confidence level, respectively, compared to DMSO+Toxin control conditions (Student’s *t-*test).

For the western blot analysis shown in Fig 1, fully differentiated mouse ES-MNs were treated with the compounds (20 μM) (Fig 2) 30 min prior to the addition of 250pM BoNT/A. For hES-MN pre-intoxication models shown in Figs 3A, 4A and 5A, fully differentiated human ES-MNs (Day 35) were treated with the titrated concentrations of compounds for 30 min and then intoxicated with either BoNT/A, BoNT/B or trypsin-activated BoNT/E (MetaBiologics, Madison, WI), and incubated at 37^°^C for 4 hr. Bafilomycin was utilized as a positive control [30]. For post-intoxication studies, cells were intoxicated with either BoNT/A, BoNT/B or trypsin-activated BoNT/E for either 30 or 60 min and then 20 μM compound was added to the cultures. Total intoxication time was kept constant at 4 hr for all immunoblotting analyses. Following intoxication, samples were prepared and BoNT mediated SNARE protein cleavage was quantified using standard immunoblotting procedures employing SNAP-25 (Covance) and VAMP-2 (R&D Systems) antibodies—as described previously [30, 36, 37]. The reported values were calculated from at least three independent experiments.

### Statistical analyses

The values are reported as the mean ± SEM and Student’s *t-*test values (P<0.05) identified statistically significant differences (GraphPad Prism version 6.01).

### Phosphatase Inhibitors

A 33 compound library of known phosphatase inhibitors were obtained from Enzo Life Sciences (Farmingdale, NY, USA).

### Assessment of BoNT/A LC inhibition using an *in vitro* HPLC-based assay

A well-characterized high-performance liquid chromatography (HPLC)-based assay was utilized to measure BoNT/A LC proteolytic activity in the presence of identified phosphatase inhibitors, as described previously [38], with slight modifications. This assay utilizes a synthetic 17 amino acid peptide that contains the BoNT/A LC cleavage site. Phosphatase inhibitors were incubated with recombinant LC and the peptide substrate in a buffer composed of 50 mM HEPES+0.2 mg/ml BSA (pH 7.4), at 37°C for 10 min in a shaking incubator. MV150, a previously identified BoNT/A LC inhibitor, was used as the positive control. The proteolytic reaction was stopped by the addition of trifluoroacetic acid and the resulting products were analyzed by reverse phase HPLC. A Shimadzu Prominence ultra fast liquid chromatography (UFLC) XR system using reverse-phase (C18:50 x 2.1 mm, 1.9 μm) chromatography was utilized for separations. The percent LC inhibition was calculated by comparing peptide cleavage obtained in the presence of phosphatase inhibitors relative to that obtained with DMSO control conditions. Three independent assays were performed with each inhibitor.

## Results

### Phosphatase inhibitors block BoNT/A activity in mouse ES-cell derived motor neurons

Previous studies have shown that SNAP-25 cleavage can be used as a phenotypic end-point to assess the effects of potential BoNT/A and /E inhibitors in cell-based functional assays [27, 39]. The rationale for quantification of SNAP-25 cleavage is linked to the observation that intact SNAP-25 is absolutely required for neuroexocytosis and that BoNT-mediated SNAP-25 cleavage is sufficient to inhibit this process [40–45]. Moreover, it is recognized that SNAP-25 is the only known intracellular target for both BoNT/A and /E [46–48]. We have previously reported a simple, fluorescence-based assay performed with mouse ES-MNs that was used to measure BoNT/A activity using BoNT/A cleavage-sensitive antibodies (BACS) [34]. This assay was used to screen a phosphatase inhibitor library for protection against BoNT/A. Mouse ES-MNs were pretreated with compounds (20 μM) for 30 min prior to the addition of 250pM BoNT/A. Following intoxication, plates were immunostained with cleavage insensitive and cleavage-sensitive SNAP-25 antibodies to measure the extent of SNAP-25 cleavage using the Li-Cor imaging assay [34]. Four compounds including NSC 663284, pentamidine, BVT 948 and 9,10-phenanthrenenequinone (9,10-PQ) were identified as potential BoNT/A intoxication inhibitors.

The four compounds were evaluated in secondary screens to confirm their protective activities against BoNT/A. Mouse ES-MNs were treated with the identified chemicals (20μM) for 30 minutes and then intoxicated with 250pM BoNT/A for an additional 4 hr. SNAP-25 cleavage was measured by western blot analyses (Fig 1). All four compounds were verified as bona fide BoNT/A antagonists as they all exhibited statistically significant (Student’s *t-*test) protection against BoNT/A (Fig 1). The chemical names and structures of the compounds are given in Fig 2.

### Phosphatase inhibitor efficacy against BoNT/A was confirmed in human ES-derived motor neurons

The finding that phosphatase inhibitors protect mouse ES-MNs against BoNT/A intoxication prompted us to test whether phosphatase inhibitor activities were relevant to BoNT intoxication in human cellular systems as well. Therefore, we tested the most effective phosphatase inhibitors for SNAP-25 protection in human ES-MNs. First, a pre-intoxication model was utilized to determine the dose-dependent effects of the compounds on BoNT/A mediated SNAP-25 cleavage. For this assay, hES-MNs were pre-treated with increasing concentrations of inhibitors (0.1 μM—20 μM) for 30 minutes and then intoxicated with 500 pM BoNT/A for 4 hr. Following intoxication, SNAP-25 cleavage was measured via western blot analysis. As expected, bafilomycin, a control compound, exhibited dose-dependent protection. Excitingly, all four inhibitors exhibited dose-dependent protection of SNAP-25 against BoNT/A in hES-MNs (Fig 3A). Consistent with the mES-MN assay, as shown in Fig 1, Pentamidine provided a qualitatively lower level of SNAP-25 cleavage protection when compared to the other compounds. Nevertheless, all four compounds antagonized BoNT/A statistically significantly (Student’s *t-*test) at 5, 10 and 20 μM concentrations (Fig 3A).

Having demonstrated pre-exposure protection against BoNT/A, the phosphatase inhibitors were next evaluated in a post-exposure model of intoxication. Human ES-MNs were intoxicated with 500 pM BoNT/A for either 30 min (Fig 3B) or 60 min (Fig 3C) to allow sufficient time for toxin internalization and processing prior to addition of the inhibitor. Phosphatase inhibitors were added at 20 μM concentration and then examined for SNAP-25 protection upon total 4 hours of BoNT intoxication. All four compounds antagonized the BoNT mediated cleavage of SNARE proteins inside human motor neurons at both 30 and 60 min post-intoxication in a statistically significant manner, with the exception of Pentamidine at 60 min post-intoxication, which trended toward, but did not reach, statistical significance (Fig 3C).

### Phosphatase inhibitors antagonize BoNT serotype B and E in a dose-dependent manner

Compounds that impact neuronal signaling processes could potentially antagonize multiple BoNT serotypes targeting different SNARE proteins if these same pathways are conserved across the intoxication process utilized by different serotypes. To test this assertion, the phosphatase inhibitors were evaluated against the distinct BoNT/B serotype that proteolyzes VAMP-2, an additional member of the SNARE complex required for neurotransmitter release [49]. Assay conditions were similar to those used in Fig 3. Increasing concentrations of inhibitor (0.1μM-20 μM) were added to hES-MNs 30 minutes prior to the addition of 1,000 pM BoNT/B. Following 4 hours of intoxication, VAMP-2 cleavage was analyzed by western blotting. Our results demonstrated that the phosphatase inhibitors were also effective at protecting VAMP-2 from the BoNT/B and that it occurred in a dose-dependent manner (Fig 4A). The phosphatase inhibitors were also evaluated in the BoNT/B post-exposure model. Human ES-MNs were intoxicated with 1000 pM BoNT/B for either 30 min or 60 min (Fig 4B and 4C respectively) and then treated with 20 μM inhibitors. Neurons were lysed upon 4 hours of intoxication and assayed for VAMP-2 cleavage. All four phosphatase inhibitors antagonized the BoNT/B mediated cleavage of VAMP-2 in a statistically significantly manner at both the 30 and 60 min time points.

To further test the potential broad-spectrum efficacy of the phosphatase inhibitors, we next evaluated their effect in protecting hES-MNs against BoNT/E. BoNT/E is an additional serotype that cleaves SNAP-25 but acting at a distinct proteolytic site that results in the removal of 26 amino acids of the protein from the C-terminal. This cleavage results in a secondary SNAP-25 specific band (~23kDa) that runs lower than the intact SNAP-25 (~25kDa) one on a SDS PAGE analysis. Human ES-MNs were treated with titrated concentrations of compounds ranging from 0.1 μM to 20 μM and then intoxicated with 500 pM trypsin-activated BoNT/E. Upon 4 hr of intoxication, samples were prepared and SNAP-25 cleavage was measured by western blotting. As shown in Fig 5A, the inhibitors were able to antagonize BoNT/E mediated SNAP-25 cleavage when used at a concentration of at least 10 μM. Next, we examined the compounds in a BoNT/E post-intoxication model. Cells were intoxicated with BoNT/E for 30 min or 60 min (Fig 5B and 5C respectively) and then treated with the compounds at 20 μM concentrations, similar to the conditions in Fig 4B and 4C, respectively. Overall, both NSC663284 and 9,10-PQ provided statistically significant protection of SNAP-25 at both the 30 and 60 min time points whereas BVT 948 was found to be efficacious at the 30 min time point. Pentamidine exhibited protection against BoNT/E under the conditions tested; however the effect was not statistically significant. Collectively, these results suggest the phosphatase inhibitors function as broad-spectrum BoNT inhibitors against serotypes A, B and E in acute pre- and post-exposure models.

### Phosphatase inhibitors do not directly inhibit BoNT/A light chain activity

This study has utilized a pharmacologic approach to interrogate the relationship between phosphatase activities and BoNT intoxication. While it is implied that the protection provided against BoNT intoxication is due to blockade of enzymatic activity of cellular phosphatases, it cannot be ruled out that protection is occurring due to the direct inhibition of the zinc-metalloprotease activity of the BoNT LC. In order to evaluate this possibility, the phosphatase inhibitors were tested for their ability to directly block BoNT/A LC activity using an *in vitro* proteolysis assay. A previously identified LC inhibitor, MV150, was utilized as a positive control, which provided an IC_50_ of 5.2+/- 3.2 μM. As shown in Table 1, all phosphatase inhibitors provided an IC_50_ of greater than 20 μM, indicating that they do not function as meaningful enzymatic inhibitors of BoNTs. Taken together, the identified inhibitors are well-characterized phosphatase inhibitors [50–55], and our data support the notion that these compounds do not directly inhibit the BoNT/A LC (Table 1), but act via modulation of host neuronal processes.

**Table 1:** Phosphatase inhibitors act on host cellular processes.

Compound	BoNT/A LC Inhibition IC_50_ (μM)
Control: MV150	5.2 +/-3.2
NSC 663284	>20
Pentamidine	>20
BVT 948	>20
9,10-Phenanthrenequinone	>20

A well-characterized HPLC-based LC inhibition assay was utilized to determine whether phosphatase inhibitors directly inhibit BoNT/A LC activity *in vitro*. MV150 is a previously identified LC inhibitor and used as a positive control. The values were calculated from three independent assays.

## Discussion

In this study, we employed both mouse and human ES- MN systems to identify small molecule multi-serotype BoNT inhibitors with neuronal efficacy. To date, the majority of BoNT drug screening studies have utilized *in vitro* proteolytic assays, and there has been very little effort examining potential drug targets using physiologically relevant cell-based systems [56]. We and others have previously established highly sensitive mouse and human ES-MN systems for BoNT studies [25, 30, 57, 58] and such biologically relevant platforms greatly enhances BoNT drug discovery as they can be used for both drug screening and biochemical studies to identify neuronal pathways that are involved in intoxication and/or recovery. Here, we used these assays to screen a library of known phosphatase inhibitors for their ability to antagonize BoNT intoxication. These studies identified four compounds that show dose-dependent protection against BoNT/A, /B and /E. Importantly, these compounds exert post-intoxication efficacy against all three serotypes, which are responsible for more than 95% of human botulism cases [15]. To best of our knowledge, this is the first study demonstrating that small molecule phosphatase inhibitors protect against BoNT intoxication in mouse and human neuronal systems.

A highly effective strategy to treat Botulism patients may involve a combination therapy in which FDA approved anti-BoNT antibodies are used to aid the toxin’s clearance before neuronal entry, coupled with cell-permeable small molecules that inhibit the LC-mediated cleavage of SNARE proteins. Toward this goal, BoNT drug development efforts have mostly focused on development of direct/competitive small molecule LC inhibitors [9]. However, despite the extensive research, no LC inhibitors have been shown to provide meaningful *in vivo* efficacy in higher mammals, demonstrating that LCs are difficult drug targets. It should also be noted that most LC drug screening assays have utilized *in vitro* approaches. Although such assays provide simple and fast drug screening platforms, they rely on the assumption that the structures of purified LCs and SNARE peptides used in these assays mimic the protein conformations that exist inside motor neurons. However, such assays fail to account for the potential effects of post-translational modifications, including phosphorylation, on the structures of the LCs and their SNARE protein targets. Additionally, other factors such as protein-protein interactions impact protein folding, which might be critical for BoNT activity. Indeed, as discussed below, it is well-established that SNARE proteins are highly regulated by phosphorylation and their conformations are also regulated through interactions with other SNARE proteins [59–61]. Therefore, *in vitro* LC drug screening assays may not be the best approximations of the true physiological conditions. Another critical issue is that LC drug development efforts have mostly focused on BoNT/A. However there is a need to develop multi-serotype BoNT inhibitors as there are at least 4 serotypes that can cause human botulism [15]. Given the remarkable substrate specificity of LCs, it seems unlikely that LC inhibitors can be effective against more than one serotype. Therefore, novel approaches are needed to develop therapeutically viable countermeasures that can be effective against multiple BoNT serotypes.

One attractive approach would involve the modulation of neuronal signaling nodes that are critical for either intoxication or recovery or both. Therefore, one main objective of the current study was to identify small molecules that act on neuronal processes with the potential to antagonize BoNT intoxication. More specifically, we sought to identify compounds that can target neuronal mechanisms and act by a means other than the inhibition of BoNT/LC proteolytic activity. It is important to note that there has been only a few compounds described as multi-serotype BoNT inhibitors, which don’t directly target the LC [9, 62, 63]. Although the sequence of events during BoNT intoxication and their SNARE substrates are generally well known [64–66], our mechanistic understanding of the neuronal pathways that play a role(s) in intoxication and/or response mechanisms remain minimal. Previous studies suggested that phosphorylation might be a key mechanism to modulate the activity of BoNTs. More specifically, it has been suggested that phosphorylation of LCs might be critical for its intracellular half-life [20–24]. Additionally, we have shown that pharmaceutically active SFK inhibitors can protect against BoNTs in motor neurons [25]. Although the function of many proteins crucial in signal transduction is tightly regulated by phosphorylation, it is still unclear whether phosphorylation events are important in BoNT intoxication.

Generally speaking, phosphatases are considered as difficult drug targets, however recent studies suggest that modulation of phosphatases with small molecules may provide viable therapeutic options [26, 67]. For example, a recent study describes a small molecule that selectively inhibits protein phosphatase 1 [68]. Similarly, recent structural studies focused on understanding the regions required for selectivity. By modifying such regions, it may be possible to tailor phosphatase inhibitors to turn them into potentially effective therapeutic agents. Additionally, some FDA approved drugs have been shown to target phosphatases after their approval. For example, Pentamidine, one of the identified BoNT inhibitors in this study, is an FDA approved drug that targets PRL phosphatases [50]. Taken together, phosphatases may be feasible drug targets [69]. With respect to the inhibitors identified in this study, it is currently not known if they may have therapeutic potential against Botulism. Nonetheless, the inhibitors can be utilized as chemical probes to elucidate the role(s) of specific phosphatases in BoNT mechanism of intoxication in motor neurons, which may provide novel drug targets to counter BoNT intoxication. Additionally, future studies can utilize these compounds as reference compounds to develop more potent inhibitors.

The identified inhibitors are overall well-characterized and their molecular targets in cells have been reported [50–55]. For example, NSC663284 is a potent and cell-permeable compound that targets the Cdc25 phosphatase family (Cdc25A, B and C) [51] although it has been suggested that Cdc25A is the primary target [70]. Cdc25 phosphatases are well-known for their role in cell division and therefore are important oncology drug target [26], but it has also been shown that Cdc25 mediated-dephosphorylation events play critical roles in post-mitotic neurons [71]. Specifically, blockade of Cdc25 inhibits neuronal cell-death [71]. Our findings suggest that modulation of Cdc25 activity by small molecules might also be critical for BoNT intoxication and/or recovery, opening new venues for future studies.

Pentamidine is an FDA approved antiprotozoal drug, which is a potent and selective inhibitor of PRL phosphatases (PRL-1, PRL-2, and PRL-3) [50]. It has been shown that both PRL-1 and-2 are present in the nervous system while PRL-3 is predominantly expressed in heart and skeletal muscle [72–74]. Importantly, Pentamidine exhibits neuroprotective abilities [75]. BVT 948 is a cell-permeable compound that exhibits activity both in culture and in animals [52]. This compound targets protein tyrosine phosphatases (PTPs) with different efficacies [52]. Although BVT 948 may be targeting several phosphatases, it exhibits greater efficacy against some PTPs [52], including PTP1B (PTPN1) and SHP-2 (PTPN11), that are drug targets for cancer, diabetes and obesity [26]. 9,10-PQ inhibits CD45 and PTP1B (PTPN1) phosphatases [53–55] and it has been shown that PTP1B plays key roles in critical cellular pathways in neurons [76, 77]. Taken together, molecular targets of the identified inhibitors have been well studied; however, it is important to recognize that the compounds might have multiple mechanisms of action and other potential targets remain to be characterized.

The precise molecular mechanism(s) underlying phosphatase inhibitor-mediated protection against BoNT/A, /B and /E is unknown but we propose three possible scenarios. First, the phosphatase inhibitors might directly modulate LC phosphorylation. As mentioned before, it has been suggested that LCs are the substrates of Src kinase *in vitro* and in culture [20–24] and the resulting phosphorylation events appear to be critical for the toxin’s stability and activity. However, further studies are needed to establish whether LCs are indeed phosphorylated in motor neurons under physiological conditions and the phosphatase inhibitors identified in this study modulate such phosphorylation events. Second, the observed antagonism against BoNTs could be simply due to off-target effects. However, observations that the inhibitors exhibit statistically significant dose-dependent protection against all three serotypes and that only 4 inhibitors in the 33 compound phosphatase inhibitor library exhibited efficacy does not support this notion. A third possibility includes phosphorylated SNARE and/or SNARE interacting proteins (rather than direct LC phosphorylation) as a target which would affect BoNT activity in neurons. Previous studies have established that kinases and phosphatases play critical roles in modulating neuroexocytosis by affecting phosphorylation states of SNARE proteins. For example, VAMP-2, SNAP-25 and Syntaxin (all BoNT targets) are phosphorylated by various kinases, including PKC [78–83]. Mechanistically, it has been shown that SNAP-25 phosphorylation affects its interaction with other SNARE proteins, which is crucial for functional SNARE formation [81, 84, 85]. Such phosphorylation events are also critical for the size and secretion of vesicles [78, 80]. Importantly, the phosphorylation state of SNAP-25 determines its compartmentalization within the cell. For example, increased SNAP-25 phosphorylation is critical for relocating cytosolic SNAP-25 to the plasma membrane, which may be necessary for BoNT activity [86, 87]. Other than the location of SNARE proteins, their conformation(s) may also be critical for BoNTs to access their cleavage sites. For example, it has been suggested that the Ser187 residue of SNAP-25 may not be available for PKC mediated phosphorylation when SNAP-25 is in a monomeric form. However, this residue is efficiently phosphorylated when SNAP-25 is in the SNARE complex [84], suggesting critical conformational changes upon protein-protein interactions. Conformational changes may be important for the LCs to access their binding site in the target proteins. Collectively, SNARE phosphorylation events are critical for exocytosis [59–61], and this implies that the equilibrium of phosphatase and kinase activity in motor neurons is an important determinant of proper neurotransmitter release. Importantly, there have been research efforts to identify phosphatases that regulate SNARE phosphorylation. For example, SNAP-25 is dephosphorylated by Protein Phosphatase 1 (PP1) and Protein Phosphatase 2A (PP2A) in both Ca^2+^-dependent and-independent manner [59, 88]. Currently, it is unknown whether the phosphorylation of SNARE proteins has functional roles in BoNT mediated cleavage of these proteins. Future studies that define the precise mechanism by which phosphatase inhibitors impact BoNT intoxication will be instrumental in developing novel therapeutics against botulism. There are significant differences between BoNT serotypes in terms of their biochemical structures, longevity and activity in neurons [47]. Nevertheless, our study identified inhibitors that protect against BoNT/A-, /B- and /E-mediated substrate cleavage both pre- and post-intoxication suggesting that BoNTs might share similar intracellular pathways for intoxication. This is also supported by the finding that the phosphatase inhibitors do not directly inhibit the BoNT/A LC (Table 1). Therefore, it is conceivable that specific phosphatase-mediated signaling pathways are important for the action of BoNTs, and that small molecule modulation of one or more of these pathways may protect SNARE proteins during intoxication of motor neurons.

## Conclusions

This study provides proof-of-concept that phosphatase inhibitors can effectively antagonize multiple BoNT serotypes in human ES-derived motor neurons in a dose-dependent manner. The identification of pan-BoNT inhibitors is noteworthy because there have been very limited efforts focusing on the development of multi-serotype BoNT inhibitors. Importantly, our results highlight the importance of host factors for antagonizing the effects of BoNTs. This is a previously understudied area in the field that clearly warrants further investigation. Finally, the inhibitors can be used as molecular tools to identify differentially regulated proteins and signaling nodes that are important for BoNT intoxication. Further mechanistic studies should help identify additional host targets useful for the development of small molecule therapeutics with broad-spectrum activity against multiple BoNT serotypes.
